# Subtle Implicit Language Facts Emerge from the Functions of Constructions

**DOI:** 10.3389/fpsyg.2015.02019

**Published:** 2016-01-28

**Authors:** Adele E. Goldberg

**Affiliations:** Department of Psychology, Princeton UniversityPrinceton, NJ, USA

**Keywords:** anaphoric one, island constraints, subject-auxiliary inversion, universal grammar, grammatical constructions

## Abstract

Much has been written about the unlikelihood of innate, syntax-specific, universal knowledge of language (Universal Grammar) on the grounds that it is biologically implausible, unresponsive to cross-linguistic facts, theoretically inelegant, and implausible and unnecessary from the perspective of language acquisition. While relevant, much of this discussion fails to address the sorts of facts that generative linguists often take as evidence in favor of the Universal Grammar Hypothesis: subtle, intricate, knowledge about language that speakers implicitly know without being taught. This paper revisits a few often-cited such cases and argues that, although the facts are sometimes even more complex and subtle than is generally appreciated, appeals to Universal Grammar fail to explain the phenomena. Instead, such facts are strongly motivated by the *functions of the constructions* involved. The following specific cases are discussed: (a) the distribution and interpretation of anaphoric *one*, (b) constraints on long-distance dependencies, (c) subject-auxiliary inversion, and (d) cross-linguistic linking generalizations between semantics and syntax.

## Introduction

We all recognize that humans have a different biological endowment than the prairie vole, the panther, and the grizzly bear. We can also agree that only humans have human-like language. Finally, we agree that adults have representations that are specific to language (for example, their representations of constructions). The question that the present volume focuses on is whether we need to appeal to representations concerning syntax that have not been learned in the usual way—that is on the basis of external input and domain-general processes—in order to account for the richness and complexity that is evident in all languages. The Universal Grammar Hypothesis is essentially a claim that we do. It asserts that certain syntactic representations are “innate,”^1^ in the sense of not being learned, and that these representations both facilitate language acquisition and constrain the structure of all real and possible human languages^2^.

I take this Universal Grammar Hypothesis to be an important empirical claim, as it is often taken for granted by linguists and it has captured the public imagination. In particular, linguists often assume that infants bring with them to the task of learning language, knowledge of noun, verb, and adjective categories, a restriction that all constituents must be binary branching, a multitude of inaudible but meaningful “functional” categories and placeholders, and constraints on possible word orders. This is what Pearl and Sprouse seem to have in mind when they note that positing Universal Grammar to account for our ability to learn language is “theoretically unappealing” in that it requires learning biases that “appear to be an order (or orders) of magnitude more complex than learning biases in any other domain of cognition” (Pearl and Sprouse, 2013, p. 24).

The present paper focuses on several phenomena that have featured prominently in the mainstream generative grammar literature, as each has been assumed to involve a purely syntactic constraint with no corresponding functional basis. When constraints are viewed as arbitrary in this way, they appear to be mysterious and are often viewed as posing a learnability challenge; in fact, each of the cases below has been used to argue that an “innate” Universal Grammar is required to provide the constraints to children *a priori*.

The discussion below aims to demystify the restrictions that speakers implicitly obey, by providing explanations of each constraint in terms of the functions of the constructions involved. That is, constructions are used in certain constrained ways and are combined with other constructions in constrained ways, because of their semantic and/or discourse functions. Since children *must* learn the functions of each construction in order to use their language appropriately, the constraints can then be understood as emerging as by-products of learning those functions. In each case, a generalization based on the communicative functions of the constructions is outlined and argued to capture the relevant facts better than a rigid and arbitrary syntactic stipulation (see also DuBois, 1987; Hopper, 1987; Michaelis and Lambrecht, 1996; Kirby, 2000; Givón, 2001; Auer and Pfänder, 2011). Thus, recognizing the functional underpinnings of grammatical phenomena allows us to account for a wider, richer range of data, and allows for an *explanation* of that data in a way that purely syntactic analyses do not.

In the following sections, functional underpinnings of the distribution and interpretation of various constructions are offered including anaphoric _one_, various long-distance dependences, subject-auxiliary inversion, and cross-linguistic linking generalizations.

## Anaphoric *one*

### Anaphoric *one*'s interpretation^3^

There are many interesting facts of language; let's consider this *one*. The last word in the previous sentence refers to an “interesting fact about language” in the first clause; it cannot refer to an interesting fact that is about something other than language. This type of observation has been taken to imply that *one* anaphora demonstrates “innate” knowledge that full noun phrases (or “DP”s) contain a constituent that is larger than a noun but smaller than a full noun phrase: an N^∕^ (*interesting fact of language* above), and, that *one* anaphora must refer to an N^∕^, and may never refer to a noun without its grammatical complement (Baker, 1978; Hornstein and Lightfoot, 1981; Radford, 1988; Lidz et al., 2003b). However, as many researchers have made clear, anaphoric *one* actually *can* refer to a noun without its complement as it does in the following attested examples from the COCA corpus (Davies, 2008; for additional examples and discussion see Lakoff, 1970; Jackendoff, 1977; Dale, 2003; Culicover and Jackendoff, 2005; Payne et al., 2013; Goldberg and Michaelis, 2015)^4^.

1. “not only would **the problem of alcoholism** be addressed, but also **the related one of violence**,” [smallest N^∕^ = *problem of alcoholism*; but *one* = “problem”]
2. “it was a **war of choice** in many ways, not **one of necessity**.” [smallest N^∕^ = *war of choice*; *one* = “war”]
3. “Turning a **sense of ostracism** into **one of inclusion** is a difficult trick. [smallest N^∕^ = *sense of ostracism*; *one* = “sense”]
4. “more a **sign of desperation** than **one of strength**” [smallest N^∕^ = *sign of desperation*; *one* = “sign”]

In each case, the “*of* phrase” (e.g., *of alcoholism* in 1) is a complement according to standard assumptions and therefore should be included in the smallest available N^∕^ that the syntactic proposal predicts *one* can refer to. Yet in each case, *one* actually refers only to the previous noun (*problem, war, sense*, and *sign*, respectively, in 1–4), and does not include the complement of the noun.

In the following section, I outline an explanation of *one's* distribution and interpretation, which follows from its discourse function. To do this, it is important to appreciate anaphoric *one*'s close relationship to numeral *one*, as described below.

### The syntactic and semantic behavior of *one* are motivated by its function

Leaving aside the wide range of linguistic and non-linguistic entities that *one* can refer to for a moment, let us consider the linguistic contexts in which *one* itself occurs. Goldberg and Michaelis (2015) observe that anaphoric *one* has the same grammatical distribution as numeral *one* (and other numerals), when the latter are used without a head noun. The only formal distinction between anaphoric *one* and the elliptical use of numeral *one* is that numeral *one* receives a sentence accent, as indicated by capital letters in Table 1, whereas anaphoric *one* must be unstressed (Goldberg and Michaelis, 2015).

**Table 1:** **Distributional contexts for anaphoric *one* and the elliptical use of cardinal *one***.

**Anaphoric *one***	**Numeral one (1)**
She asked for one.	She asked for ONE
She got a blue one.	She got a mere ONE.
She only wanted that one.	She only wanted that ONE.
She was one of a group.	She was ONE of a group.

The two differ only in that only numeral one receives a sentence accent and asserts the quantity “1.”

The difference in accent between cardinal and anaphoric *one* reflects a key difference in their functions. Whereas cardinal *one* is used to assert the quantity “1,” anaphoric *one* is used when quality or existence—not quantity—is at issue. That is, if asked about quantity as in (5), a felicitous response (5a) involves cardinal *one*, which is necessarily accented (5a; cf. 5b). If the *type of entity* is at issue as in (6), then anaphoric *one*, which is necessarily unaccented, is used (6b; cf. 6a):

5. Q: *How many* dogs does she have?
a. She has (only) ONE.     (cardinal ONE)
b. #She has a big one.     (anaphoric one)
6. Q: *What kind of* dog does she have
a. #She has (only) ONE     (cardinal ONE)
b. She has a BIG one.     (anaphoric one).

It is this fact, that anaphoric *one* is used when quality and not quantity is at issue, that explains why anaphoric *one* so readily picks out an entity, recoverable in the discourse context, that often corresponds to an N^∕^: anaphoric *one* often refers to a noun and its complement (or modifier) because the complement or modifier supplies the quality. But the quality can be expressed explicitly as it is in (6b; with *big*) or in (1–4) with the overt complement phrases^5^. If existence (and not quality *or* quantity) is at issue, anaphoric *one* can refer to a full noun phrase as in (7):

7. [Who wants a drink?] I'll take one.

Thus, given the fact that anaphoric *one* exists in English, its semantic relationship to cardinal numeral *one* predicts its distribution and interpretation. Anaphoric *one* is used when the quality or existence of an entity evoked in the discourse—not its cardinality—is relevant.

The only additional fact that is required is a representation of the plural form, *ones*, and both the form and the function of *ones* is motivated because *ones* is a lexicalized extension of anaphoric *one* (Goldberg and Michaelis, 2015). *Ones* differs from anaphoric *one* only in being plural both formally and semantically; like singular anaphoric *one*, plural *ones* evokes the quality or existence and not the cardinality of a type of entity recoverable in context.

There are several lessons that can be drawn from this simple case. First, if we are too quick to assume a purely syntactic generalization without careful attention to attested data, it is easy to be led astray. Moreover, it is important to recognize relationships among constructions. In particular, anaphoric *one* is systematically related to numeral *one*, and a comparison of the functional properties of these closely related forms serves to explain their distributional properties.

There remain interesting questions about how children learn the function of anaphoric *one*. But once we acknowledge that children *do* learn its function—and they must in order to use it in appropriate discourse contexts—there is nothing mysterious about its formal distribution.

## Constraints on long distance dependencies

### The basic facts

Most languages allow constituents to appear in positions other than their most canonical ones, and sometimes the distance between a constituents' actual position and its canonical position can be quite long. For example, when questioned, the phrase *which/that coffee* in (8) is not where it would appear in a canonical statement; instead, it is positioned at the front of the sentence, and there is a *gap* (indicated by “____”) where it would normally appear.

8. Which coffee did Pam say Sam likes ____better than tea?
(cf. Pam said Sam likes that coffee better than tea.)

This type of relationship is often discussed as if the constituent “moved” or was “extracted” from its canonical position, although no one has believed since Fodor et al. (1974) that the movement is anything more than a metaphor. I use more neutral terminology here and refer to the relation between the actual position and the canonical position as a long-distance dependency (LDD).

There are several types of LDD constructions including wh-questions, the topicalization construction, cleft constructions, and relative clause constructions. These are exemplified in Table 2.

**Table 2:** **Examples of long distance dependency (LDD) constructions: constructions in which a constituent appears in a fronted position instead of where it would canonically appear**.^6^

Wh-questions	What did Pam say Sam likes ___better than tea?
Topicalization construction	That coffee, Pam said Sam likes ___better than tea.
It-cleft construction	It was that coffee that Pam said Sam likes ___better than tea.
Relative Clause construction	She tasted the coffee that Pat said Sam likes __better than tea.

Ross (1967) long ago observed that certain other types of constructions resist containing the gap of a LDD. That is, certain constructions are “islands” from which constituents cannot escape. Combinations of an “island construction” with a LDD construction result in ill-formedness (see Table 3):

**Table 3:** **Examples of island constructions: constructions that resist containing the gap in a LDD (Ross, 1967)**.

??Who did [that she hit _] was horrible? (cf. [That she hit him] was horrible.)	Subjects
??Who did she see [the boy who met __]?	
(cf. She saw [the boy who met Sally])	(Most) complex noun phrases
??What did she read [the letter that was about__]? (cf. She read [the letter that was about mountains])	
??Who did she mumble/deny [he liked__]? (cf. She mumbled/denied [he liked Katherine]).	Clausal complements of manner of speaking and factive verbs
??Who did she eat spaghetti [while talking to __on the phone]? (cf. She ate spaghetti [while talking to Jenny on the phone]).	(Certain) adjuncts

### A clash between the functions of LDD constructions and the functions of island constructions

Several researchers have observed that information structure plays a key role in island constraints (Takami, 1989; Deane, 1991; Engdahl, 1997; Erteschik-Shir, 1998; Polinsky, 1998; Van Valin, 1998; Goldberg, 2006, 2013; Ambridge and Goldberg, 2008). Information structure refers to the way that information is “packaged” for the listener: constituents are topical in the discourse, part of the potential focus domain, or are backgrounded or presupposed (Halliday, 1967; Lambrecht, 1994). Different constructions that convey “the same thing,” typically exist in a given language in order to provide different ways of packaging the information, and thus information structure is perhaps the most important reason why languages have alternative ways to say the “same” thing. As explained below, the ill-formedness of island effects arises essentially from a clash between the function of the LDD construction and the function of the island construction. First, a few definitions are required.

The focus domain is that part of a sentence that is asserted. It is thus “one kind of emphasis, that whereby the speaker marks out a part (which may be the whole) of a message block as that which he wishes to be interpreted as informative” Halliday (1967: 204). Similarly Lambrecht (1994: 218) defines the focus relation as relating “the pragmatically non-recoverable to the recoverable component of a proposition [thereby creating] a new state of information in the mind of the addressee.” What parts of a sentence fall within the focus domain can be determined by a simple negation test: when the main verb is negated, only those aspects of a sentence within the potential focus domain are negated. Topics, presupposed constituents, constituents within complex noun phrases, and parenthetical remarks are not part of the focus domain, as they are not negated by sentential negation:^7^

9. Pam, as I told you before, didn't sell the book to the man she just met.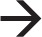 negates that the book was sold; does not negate that she just met a man or that the speaker is repeating herself.

It has long been observed that the gap in a LDD construction is typically within the potential focus domain of the utterance (Takami, 1989; Erteschik-Shir, 1998; Polinsky, 1998; Van Valin, 1998; see also Morgan, 1975): this predicts that topics, presupposed constituents, constituents within complex noun phrases, and parentheticals are all island constructions and they are (see previous work and Goldberg, 2013 for examples).

It is necessary to expand this view slightly by defining backgrounded constituents to include everything in a clause except constituents within the focus domain *and the subject*. Like the focus domain, the subject argument is part of what is made prominent or foregrounded by the sentence in the given discourse context, since the subject argument is the default topic of the clause or what the clause is “about” (MacWhinney, 1977; Chafe, 1987; Langacker, 1987; Lambrecht, 1994). That is, a clausal topic is a “matter of [already established] current interest which a statement is about and with respect to which a proposition is to be interpreted as relevant” (Michaelis and Francis, 2007: 119). The topic serves to contextualize other elements in the clause (Strawson, 1964; Kuno, 1976; Langacker, 1987; Chafe, 1994). We can now state the restriction on LDDs succinctly:

⋆ Backgrounded constituents cannot be “extracted” in LDD constructions (Backgrounded Constituents are Islands; Goldberg, 2006, 2013).

The claim in ⋆ entails that only elements within the potential focus domain *or* the subject are candidates for LDDs. Notice that constituents properly contained *within* the subject argument are backgrounded in that they are not themselves the primary topic, nor are they part of the focus domain. Therefore, subjects are “islands” to extraction.

*Why* should ⋆ hold? The restriction follows from a clash of the functions of LDD constructions and island constructions. As explained below: a referent cannot felicitously be both discourse-prominent (in the LDD construction) and backgrounded in discourse (in the island construction). That is, LDD constructions exist in order to position a particular constituent in a discourse-prominent slot; island constructions ensure that the information that they convey is backgrounded in discourse. It is anomalous for an argument, which the speaker has chosen to make prominent by using a LDD construction, to correspond to a gap that is within a backgrounded (island) construction.

What is meant by a discourse-prominent position? The wh-word in a question LDD is a classic focus, as are the fronted elements in “cleft” constructions, another type of LDD. The fronted argument in a topicalization construction is a newly established topic (Gregory and Michaelis, 2001)^8^. Each of these LDD constructions operates at the sentence level and the main clause topic and focus are classic cases of discourse-prominent positions.

The relative clause construction is a bit trickier because the head noun of a relative clause—the “moved” constituent—is not necessarily the main clause topic or focus, and so it may not be prominent in the general discourse. For this reason, it has been argued that relative clauses involve a case of recycling the formal structure and constraints that are motivated in the case of questions to apply to a distinct but related case: relative clauses (Polinsky, 1998). But in fact, the head noun in a relative clause construction is prominent when it is considered *in relation to* the relative clause itself: the purpose of a relative clause is to identify or characterize the argument expressed by the head noun. In this way, the head noun should not correspond to a constituent that is backgrounded within the relative clause. Thus, there is a clash for the same reason that sentence level LDD constructions clash with island constructions, except that what is prominent and what is backgrounded *is relative to* the content of the NP: the head noun is prominent and any island constructions within the relative clause are backgrounded.

We should expect the ill-formedness of LDDs to be gradient and degrees of ill-formedness are predicted to correspond to degrees of backgroundedness, when other factors related to frequency, plausibility, and complexity are controlled for. This idea motivated an experimental study of various clausal complements, including “bridge” verbs, manner-of-speaking verbs, and factive verbs and exactly the expected correlation was found (Ambridge and Goldberg, 2008): the degree of acceptability of extraction showed a strikingly strong inverse correlation with the degree of backgroundedness of the complement clause—which was operationalized by judgments on a negation test. Thus, the claim is that each construction has a function and that constructions are combined to form utterances; constraints on “extraction” arise from a clash of discourse constraints on the constructions involved.

The functional account predicts that certain cases pattern as they do, even though they are exceptional from a purely syntactic point of view (see also Engdahl, 1997). These include the cases in Table 4. Nominal complements of indefinite “picture nouns” fall within the focus domain, as do certain adjuncts, while the recipient argument of the double object construction, as a secondary topic, does not (see Goldberg, 2006, 2013 for discussion). Therefore, the first two cases in Table 2 are predicted to allow LDDs while the final case is predicted to resist LDDs^9^. No special assumptions or stipulations are required.

**Table 4:** **Cases that follow from an information structure account, but not from an account that attempts to derive the restrictions from configurations of syntactic trees**.

Who did she take [a picture of __]? (cf. She took [a picture of Sally])	Reduced relative clauses that are within the focus domain (e.g., “picture NPs”) are not islands; those that are not within the focus domain are islands.
Who did she wait in line [in order to see __]?	Non-backgrounded adjuncts
(cf. She waited in line [in order to see U2]).	
??Who did she give the book?^10^ (cf. She gave Aliza the book.) (cf. also, Who did she give the book to?)	Backgrounded (as the secondary topic) recipient argument of the double object construction

There is much more to say about island effects (see e.g., Sprouse and Hornstein, 2013). The hundreds of volumes written on the subject cannot be properly addressed in a short review such as this. The goal of this section is to suggest that a recognition of the functions of the relevant constructions involved can explain which constructions are islands and why; much more work is required to explore whether this proposal accounts for each and every LDD construction in English and other languages.

## Subject auxiliary inversion (SAI)

### SAI's distribution

Subject-auxiliary inversion (e.g., *is this it?*) has a distribution that is quite unique to English. In Old English, it followed a more general “verb second” pattern, which still exists in Germanic and a few other languages. But English changed, as languages do, and today, subject-auxiliary inversion requires an *auxiliary* verb and is restricted to a limited range of constructions, enumerated in (10–17):

10. Did she go?                                                                         Y/N questions
        Where did she go?                                         (non-subject) WH-questions
11. Had she gone, they would be here by now.        Counterfactual conditionals
12. Seldom had she gone there.                                     Initial negative adverbs
13. May a million fleas infest his armpits!                                   Wishes/Curses
14. He was faster at it than was she.                                           Comparatives
15. Neither do they vote.                                                     Negative conjunct
16. Boy did she go, or what?!                                                      Exclamatives
17. So does he.                                                  Positive elliptical conjunctions

When SAI is used, the entire subject argument appears after the first main clause auxiliary as is clear in a comparison of (18a) and (18b):

18. a.Has the girl who was in the back of the room had enough to eat? (inverted).
b. The girl who was in the back of the room has had enough to eat. (non-inverted).

Notice that the very first auxiliary in the corresponding declarative sentence (*was*) cannot be inverted (see 19a), nor can the second (or other) main clause auxiliary (see 19b).

19. a. ^*^Was the girl who in the back of the room has had enough to eat? (only the main clause auxiliary can be inverted).
b. ^*^Had the girl who was in the back of the room has enough to eat? (only the *first* main clause auxiliary can be inverted).

Thus, the generalization at issue is that the first auxiliary in the full clause containing the subject is inverted with the entire subject constituent.

SAI occurs in a range of constructions in English and each one has certain unique constraints and properties (Fillmore, 1999; Goldberg, 2009); for example, in the construction with negative adverbs (e.g., 12), the adverb is positioned clause initially; curses (e.g., 13) are quite particular about which auxiliary may be used (*May a million fleas invest your armpits. vs*.^*^Might/will/shall a million fleas invest your armpits!); and inversion in comparatives (e.g., 14) is restricted to a formal register. Thus, any descriptively adequate account of SAI in English must make reference to these properties of individual constructions.

The English constructions evolved diachronically from a more general constraint which still operative in German main clauses. But differences exist across even these closely related languages. The German constraint applies to main verbs, while English requires an auxiliary verb, and in English the auxiliary is commonly in first not second position (e.g., *did I get that right*?). Also, verb-second in German is a main clause phenomenon, but in English, SAI is possible in embedded clauses as well (20–21):

20. “And Janet, do you think that had he gotten a diagnosis younger, it would have been a different outcome?” (COCA)
21. “Many of those with an anti-hunting bias have the idea that were it not for the bloodthirsty human hunter, game would live to ripe old age” (COCA)

Simple recurrent connectionist networks can learn to invert the correct auxiliary on the basis of simpler input that children uncontroversially receive (Lewis and Elman, 2001). This model is instructive because it is able to generalize correctly to produce complex questions (e.g., *Is the man who was green here*?), after receiving training on simple questions and declarative statements with a relative clause. The network takes advantage of the fact that both simple noun phrases (*the boy*) and complex noun phrases (*The boy who chases dogs*) have similar distributions in the input (see also Pullum and Scholz, 2002; Reali and Christiansen, 2005,^11^; Ambridge et al., 2006; Rowland, 2007; Perfors et al., 2011).

*The reason* simple and complex subjects have similar distributions is that the subject is a coherent semantic unit, typically referring to an entity or set of entities. For example, in (22a–c), *he, the boy*, and *the boy in the front row*, all identify a particular person and each sentence asserts that the person in question is tall.

22. a.He is tall.
b. The boy is tall.
c.The boy who sat in front of me is tall.

Thus the distributional fact that is sufficient for learning the key generalization is that subjects, whether simple or complex, serve the same function in sentences.

We might also ask *why* SAI is used in the range of constructions it is, and why these constructions use this formal feature instead of placing the subject in sentence-final position or some other arbitrary feature. Consider the function of the first auxiliary of the clause containing the subject. This auxiliary indicates tense and number agreement (23), but an auxiliary is not required for these functions, as the main verb can equally well express them (24).

23. a. She did say.
b. They do say.
24. a. She said.
b. They say.

The first auxiliary of the clause containing the subject obligatorily serves a different purpose related to negative or emphasized positive polarity (Langacker, 1991). That is, if a sentence is negated, the negative morpheme occurs immediately after—often cliticized to—the first auxiliary of the clause that contains the subject (25):

25. She hadn't been there.

And if positive polarity is emphasized, it is the first auxiliary that is accented (26):

26. She HAD been there. (cf. She had been there).

If the corresponding simple positive sentence does not contain an auxiliary, the auxiliary verb *do* is drafted into service (27):

27. a. She DID swim in the ocean.
b. She did not swim in the ocean.
c. She didn't swim in the ocean.
(cf. She swam in the ocean).

Is it a coincidence that the first auxiliary of the main clause that contains the subject conveys polarity? Intriguingly, most SAI constructions offer different ways to implicate a negative proposition, or at least to avoid asserting a simple positive one (Brugman and Lakoff, 1987; Goldberg, 2006)^12^. For example, yes/no questions ask whether or not the proposition is true; counterfactual conditionals deny that the antecedent holds; and the inverted clause in a comparative can be paraphrased with a negated clause as in (28):

28. He was faster than was she. 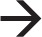 She was not as fast as he was.

Exclamatives have the form of rhetorical yes/no questions, and in fact they commonly contain tag questions (e.g., *Is he a jerk, or what?!*) (Goldberg and Giudice, 2005). They also have the pragmatic force of emphasizing the positive polarity, which we have seen is another function of the first auxiliary. Likewise, the positive conjunction (*so did she*) emphasizes positive polarity as well.

Thus the form of SAI in English is motivated by the functions of the vast majority of SAI constructions: in order to indicate non-canonical polarity of a sentence—either negative polarity or emphasized positive polarity—the auxiliary required to convey polarity is inverted. Once the generalization is recognized to be iconic in this way, it becomes much less mysterious both from a descriptive and an acquisition perspective.

There is only one case where SAI is used without implicating either negative polarity or emphasizing positive polarity: non-subject wh-questions. This case appears to be an instance of recycling a formal pattern for use with a construction that has a related function to one that is directly motivated (see also Nevalainen, 1997). In particular, wh-questions have a function that is clearly related to yes/no questions since both are questions. But while SAI is directly motivated by the non-positive polarity of yes/no questions, this motivation does not extend to wh-questions (also see Goldberg, 2006 and Langacker, 2012 for a way to motivate SAI in wh-questions more directly). Nonetheless, to ignore the relationship between the function of the first auxiliary as an indicator of negative polarity or emphasized positive polarity, and the functions of SAI constructions, which overwhelmingly involve exactly the same functions, is to overlook an *explanation* of the construction's formal property and its distribution. Thus, we have seen that the fact that the subject is treated as a unit (so that any auxiliary within the subject is irrelevant) is not mysterious once we recognize that it is a semantic unit. Moreover, the fact that it is the *first* auxiliary of the clause that is inverted is motivated by the functions of the constructions that exhibit SAI.

## Cross-linguistic generalizations about the linking between semantics and syntax

The last type of generalization considered here is perhaps the most straightforward. There are certain claims about how individual semantic arguments are mapped to syntax that have been claimed to require syntactic stipulation, but which follow straightforwardly from the semantic functions of the arguments.

Consider the claimed universal that the number of semantic arguments equals the number of overt complements expressed (the “θ criterion”; see also Lidz et al., 2003a). While the generalization holds, roughly, in English, it does not in many—perhaps the majority—of the world's languages, which readily allow recoverable or irrelevant arguments to be omitted. Even in English, particular constructions circumvent the general tendency. For example, short passives allow the semantic agent or causer argument to be unexpressed (e.g., *The duck was killed*), and the “deprofiled object construction” allows certain arguments to be omitted because they are irrelevant (e.g., *Lions only kill at night)*. (Goldberg, 2000). Thus, the original syntactic claim is too strong. A more modest, empirically accurate generalization is captured by the following:

Pragmatic Mapping Generalization (Goldberg, 2004):

- The referents of linguistically expressed arguments are interpreted to be *relevant* to the message being conveyed.
- Any semantic participants in the event being conveyed that are *relevant* and *non-recoverable* from context must be overtly indicated.

The pragmatic mapping generalization makes use of the fact that language is a means of communication and therefore requires that speakers say as much as is necessary but not more (Paul, 1889; Grice, 1975). Note that the pragmatic generation does not make any predictions about semantic arguments that are recoverable or irrelevant. This is important because, as already mentioned, languages and constructions within languages treat those arguments variably.

Another general cross-linguistic tendency is suggested by Dowty (1991), who proposed a linking generalization that is now widely cited as capturing the observable (i.e., surface) cross-linguistic universals about how syntactic relations and semantic arguments are linked. Dowty argued that in simple active clauses, *if* there both a subject and an object, *and if* there is an agent-like semantic argument and an undergoer-like semantic argument, then the agent will be expressed by the subject, and the undergoer will be expressed by the direct object (see also Van Valin, 1990). Agent-like entities are entities that are volitional, sentient, causal or moving, while undergoers are those arguments that undergo a change of state, are causally affected or are stationary. Dowty further observed that his generalization is violated in syntactically ergative languages, which are quite complicated and do not neatly map the agent-like argument to a subject. In fact, there are no syntactic tests for subjecthood that are consistent across languages so there is no reason to assume that the grammatical relation of subject is universal (Dryer, 1997).

At the same time, there does exist a more modest “linking” generalization that is accurate: actors and undergoers are generally expressed in prominent syntactic slots (Goldberg, 2006). This simpler generalization, which I have called the *salient-participants-in-prominent-slots* generalization has the advantage that it accurately predicts that an actor argument without an undergoer, and an undergoer without an actor are also expressed in prominent syntactic positions.

The tendency to express salient participants in prominent slots follows from well-documented aspects of our general attentional biases. Humans' attention is naturally drawn to agents, even in non-linguistic tasks. For example, visual attention tends to be centered on the agent in an event (Robertson and Suci, 1980). Speakers also tend to adopt the perspective of the agent of the event (MacWhinney, 1977; Hall et al., 2013). Infants as young as 9 months have been shown to attribute intentional behavior even to inanimate objects that have appropriate characteristics (e.g., motion, apparent goal-directedness) (Csibra et al., 1999). That is, even, pre-linguistic infants attend closely to the characteristics of agents (volition, sentience, and movement) in visual as well as linguistic tasks.

The undergoer in an event is also attention-worthy, as it is generally the endpoint of a real or metaphorical force (Langacker, 1987; Talmy, 1988; Croft, 1991). The tendency to attend closely endpoints of actions that involve a change of state exists even in 6 month old infants (Woodward, 1998), and we know that the effects of actions play a key role in action-representations both in motor control of action and in perception (Prinz, 1990, 1997). For evidence that undergoers are salient in non-linguistic tasks, see also Csibra et al. (1999); Bekkering et al. (2000); Javanovic et al. (2007). For evidence that endpoints or undergoers are salient in linguistic tasks, see Regier and Zheng (2003); Lakusta and Landau (2005), and Lakusta et al. (2007). Thus, the observation that agents and undergoers tend to be expressed in prominent syntactic positions is explained by general facts about human perception and attention.

Other generalizations across languages are also amenable to functional explanations. There is a strong universal tendency for languages to have some sort of construction that can reasonably be termed a “passive.” But these passive constructions only share a general function: they are constructions in which the topic and/or agent argument is essentially “demoted,” appearing optionally or not at all. In this way, passive constructions offer speakers more flexibility in how information is packaged. But whether or which auxiliary appears, whether a given language has one, two, or three passives, whether or not intransitive verbs occur in the pattern, and whether or how the demoted subject argument is marked, all *differ* across different languages (Croft, 2001), and certain languages such as Choctaw do not seem to contain any type of passive (Van Valin, 1980). That is the only robust generalization about passive depends on its function and is very modest: most, but not all languages, have a way to express what is normally the most prominent argument in a less prominent position.

## Conclusion

When it was first proposed that our knowledge of language was so complex and subtle and that the input was so impoverished that certain syntactic knowledge must be given to us *a priori*, the argument was fairly compelling (Chomsky, 1965). At that time, we did not have access to large corpora of child-directed speech so we did not realize how massively repetitive the input was; nor did we have large corpora of children's early speech, so we did not appreciate how closely children's initial productions reflect their input (see e.g., Mintz et al., 2002; Cameron-Faulkner et al., 2003). We also had not yet fully appreciated how statistical learning worked, nor how powerful it was (e.g., Saffran et al., 1996; Gomez and Gerken, 2000; Fiser and Aslin, 2002; Saffran, 2003; Abbot-Smith et al., 2008; Wonnacott et al., 2008; Kam and Newport, 2009). Connectionist and Bayesian modeling had not yet revealed that associative learning and rational inductive inferences could be used to address many aspects of language learning (see e.g., Elman et al., 1996; Perfors et al., 2007; Alishahi and Stevenson, 2008; Bod, 2009). The important role of language's function as a means of communication was widely ignored (but see e.g., Lakoff, 1969; Bolinger, 1977; DuBois, 1987; Langacker, 1987; Givón, 1991). Finally, the widespread recognition of emergent phenomena was decades away (e.g., Karmiloff-Smith, 1992; Lander and Schork, 1994; Elman et al., 1996). Today, however, armed with these tools, we are able to avoid the assumption that all languages must be “underlyingly” the same in key respects or learned via some sort of tailor-made “Language Acquisition Device” (Chomsky, 1965). In fact, if Universal Grammar consists only of recursion via “merge,” as Chomsky has proposed (Hauser et al., 2002), it is unclear how it could even begin to address the purported poverty of the input issue in any case (Ambridge et al., 2015).

Humans are unique among animals in the impressive diversity of our communicative systems (Dryer, 1997; Croft, 2001; Tomasello, 2003:1; Haspelmath, 2008; Evans and Levinson, 2009; Everett, 2009). If we assume that all languages share certain important formal parallels “underlyingly” due to a tightly constrained Universal Grammar, except perhaps for some simple parameter settings, it would seem to be an unexplained and maladaptive feature of languages that they involve such rampant superficial variation. In fact, there are cogent arguments against positing innate, syntax-specific, universal knowledge of language, as it is biologically and evolutionarily highly implausible (Christiansen and Kirby, 2003; Chater et al., 2009; Christiansen and Chater, 2016).

Instead, what makes language possible is a certain combination of prerequisites for language, including our pro-social motivation and skill (e.g., Hermann et al., 2007; Tomasello, 2008); the general trade off between economy of effort and maximization of expressive power (e.g., Levy, 2008; Futrell et al., 2015; Kirby et al., 2015; Kurumada and Jaeger, 2015); the power of statistical learning (Saffran et al., 1996; Gomez and Gerken, 2000; Saffran, 2003; Wonnacott et al., 2008; Kam and Newport, 2009); and the fact that frequently used patterns tend to become conventionalized and abbreviated (Heine, 1992; Dabrowska, 2004; Bybee et al., 1997; Verhagen, 2006; Traugott, 2008; Bybee, 2010; Hilpert, 2013; Traugott and Trousdale, 2013; Christiansen and Chater, 2016).

While these prerequisites for language are highly pertinent to the discussion of whether we need to appeal to a Universal Grammar, the present paper has attempted to address a different set of facts. Many generative linguists take the existence of subtle, intricate, knowledge about language that speakers implicitly know without being taught as evidence in favor of the Universal Grammar Hypothesis. By examining certain of these well-studied such cases, we have seen that, while the facts are sometimes even more complex and subtle than is generally appreciated, they do not require that we resort to positing syntactic structures that are unlearned. Instead, these cases are explicable in terms of the *functions of the constructions* involved. That is, the constructionist perspective views intricate and subtle generalizations about language as emerging on the basis of domain-general constraints on perception, attention, and memory, *and* on the basis of the functions of the learned, conventionalized constructions involved. This paper has emphasized the latter point.

Constructionists recognize that languages are not unconstrained in their variation and that various systematic patterns recur in unrelated languages. While certain generalizations follow from domain-general processing constraints (see e.g., McRae et al., 1998; Hawkins, 1999; Futrell et al., 2015), this paper as argued that many constraints and generalizations follow from the functions of the constructions involved. That is, speakers can combine conventional constructions in their language on the fly to create new utterances, but the functions of each of the constructions involved must be respected. This allows speakers to use language in dynamic, but delimited ways.

## Author contributions

AG wrote the paper in its entirety with appropriately cited references.

### Conflict of interest statement

The author declares that the research was conducted in the absence of any commercial or financial relationships that could be construed as a potential conflict of interest.

## Footnotes

^1^I put the term “innate” in quotes because the term lacks an appreciation of the typically complex interactions between genes and the environment before and after birth (see Deák, 2000; Blumberg, 2006; Karmiloff-Smith, 2006 for relevant discussion).

^2^*Universal Grammar* seems to mean different things to different researchers. In order for it to be consistent with its nomenclature and its history in the field, I take the Universal Grammar Hypothesis to claim that there exists some sort of universal but unlearned (“innate”) knowledge of language that is specific to grammar.

^3^This section is based on Goldberg and Michaelis (2015), which contains a much more complete discussion of anaphoric *one* and its relationship to numeral *one* (and other numerals).

^4^A version of the first sentence also allows *one* to refer to an interesting *fact* that is not about language: a. There are many interesting facts of language, but let's consider this *one* about music.

^5^To fully investigate the range of data that have been proposed to date in the literature, judgment data should be collected in which contexts are systematically varied to emphasize definiteness, quality, existence and cardinality.

^6^Other LDD constructions include comparatives (Bresnan, 1972; Merchant, 2009) and “tough” movement constructions (Postal and Ross, 1971) which should fall under the present account as well; more study is needed to investigate these cases systematically from the current perspective (see Hicks (2003); Sag (2010); for discussion).

^7^Backgrounded constituents can be negated with “metalinguistic” negation, signaled by heavy lexical stress on the negated constituent (*I didn't read the book that Maya gave me because she didn't GIVE me any book!*). But then metalinguistic negation can negate anything at all, including intonation, lexical choice, or accent. Modulo this possibility, the backgrounded constituents of a sentence are not part of what is asserted by the sentence.

^8^The present understanding of discourse prominence implicitly acknowledges the notions of topic and focus are not opposites: both allow for constituents to be interpreted as being prominent (see, e.g., Arnold, 1998: for experimental and corpus evidence demonstrating the close relationship between topic and focus). This makes sense once we realize that one sentence's focus is often the next sentence's topic.

^9^Cross linguistic work is needed to determine whether secondary topics generally resist LDDs as is the case in the English double-object construction, or whether the dispreference is only detectable when an alternative possibility is available, as in English, where questioning the recipient of the *to*-dative is preferred (see note 10).

^10^Support for this judgment comes from the fact that questions of the recipient of the *to*-dative outnumber those of the recipient of the double-object construction in corpus data by a factor of 40 to 1 (Goldberg, 2006: 136).

^11^See Kam et al. (2008) for discussion of the difficulties of using only bi-grams. Since we assume that meaningful units are combined to form larger meaningful units, resulting in hierarchical structure, this critique does not undermine the present proposal.

^12^Labov (1968) discusses another SAI construction used in AAVE, which requires a negated auxiliary (e.g., *Can't nobody go there*.).
